# Best practices in scaling digital health in low and middle income countries

**DOI:** 10.1186/s12992-018-0424-z

**Published:** 2018-11-03

**Authors:** Alain B. Labrique, Christina Wadhwani, Koku Awoonor Williams, Peter Lamptey, Cees Hesp, Rowena Luk, Ann Aerts

**Affiliations:** 1 Department of International Health, Johns Hopkins Bloomberg School of Public Health, 615 N Wolfe St, Rm E5543, Baltimore, MD 21205 USA; 2 Novartis Foundation, Basel, Switzerland; 3 Ghana Health Service, Accra, Ghana; 4 London School of Hygiene & Tropical Medicine, London, UK; 5 Family Health International 360, London, UK; 6 PharmAccess Foundation, Amsterdam, The Netherlands; 7 Dimagi South Africa, Cape Town, South Africa

**Keywords:** Digital health, Health policy, Health system, Intervention, low and middle income countries, mHealth, Programme sustainability

## Abstract

Healthcare challenges in low and middle income countries (LMICs) have been the focus of many digital initiatives that have aimed to improve both access to healthcare and the quality of healthcare delivery. Moving beyond the initial phase of piloting and experimentation, these initiatives are now more clearly focused on the need for effective scaling and integration to provide sustainable benefit to healthcare systems.

Based on real-life case studies of scaling digital health in LMICs, five key focus areas have been identified as being critical for success. Firstly, the intrinsic characteristics of the programme or initiative must offer tangible benefits to address an unmet need, with end-user input from the outset. Secondly, all stakeholders must be engaged, trained and motivated to implement a new initiative, and thirdly, the technical profile of the initiative should be driven by simplicity, interoperability and adaptability. The fourth focus area is the policy environment in which the digital healthcare initiative is intended to function, where alignment with broader healthcare policy is essential, as is sustainable funding that will support long-term growth, including private sector funding where appropriate. Finally, the extrinsic ecosystem should be considered, including the presence of the appropriate infrastructure to support the use of digital initiatives at scale.

At the global level, collaborative efforts towards a less-siloed approach to scaling and integrating digital health may provide the necessary leadership to enable innovative solutions to reach healthcare workers and patients in LMICs. This review provides insights into best practice for scaling digital health initiatives in LMICs derived from practical experience in real-life case studies, discussing how these may influence the development and implementation of health programmes in the future.

## Background

A wide range of digital health initiatives have been piloted in response to specific healthcare challenges in low and middle income countries (LMICs). These leverage ubiquitous technologies such as mobile phone networks and devices, combined with increasingly sophisticated national level health information systems and information exchanges. To move beyond the pilot stage, the focus of digital health initiatives has been shifting towards scalability, integration and sustainability, with the goal of improving both health system processes and health outcomes. The emphasis on scale and sustainability is driven in part by the desire of LMIC governments, partner agencies and the private sector to invest in initiatives that provide measurable, long-term impact on the delivery of health programmes. Practical experience of scaling and integrating digital health in LMICs has highlighted intrinsic criteria and enabling conditions that can support new initiatives to reach scale and become fully integrated in healthcare systems. This review describes key considerations for scaling digital health solutions in LMICs, distilled from real-life case studies discussed at a Digital Health Dialogue held in Ghana.

To assess successful scaling of digital health initiatives, it is first necessary to define ‘scale’, considering the perspectives of end-users, patients, healthcare policy makers and investors, and the unmet needs within the relevant healthcare systems. One definition of successful scale could be when a digital solution is not seen as a separate activity, but is incorporated seamlessly within the healthcare system. Besides integration, other possible aspirations for scale proposed by the Program for Appropriate Technology in Health (PATH) include sustainability, with respect to funding and government support, and the ability to replicate, refine and improve over time (Table 1) [1].

**Table 1:** Aspirations for investment in and scaling of digital healthcare innovations [1]

• Triggered and selected according to the needs of the health system	
• Mandated and driven by the Ministry of Health (or country-specific equivalent)	
• Enabled by committed, long-term funding and robust programme management so solutions have time and support to iterate, evolve and embed into existing systems and practices	
• Built around realistic, long-term funding models	
• Integrated into existing national platforms	
• Selected and designed to conform to agreed standards	
• Designed and implemented with the participation of the end-users and long-term implementers	

**Fig. 1:**
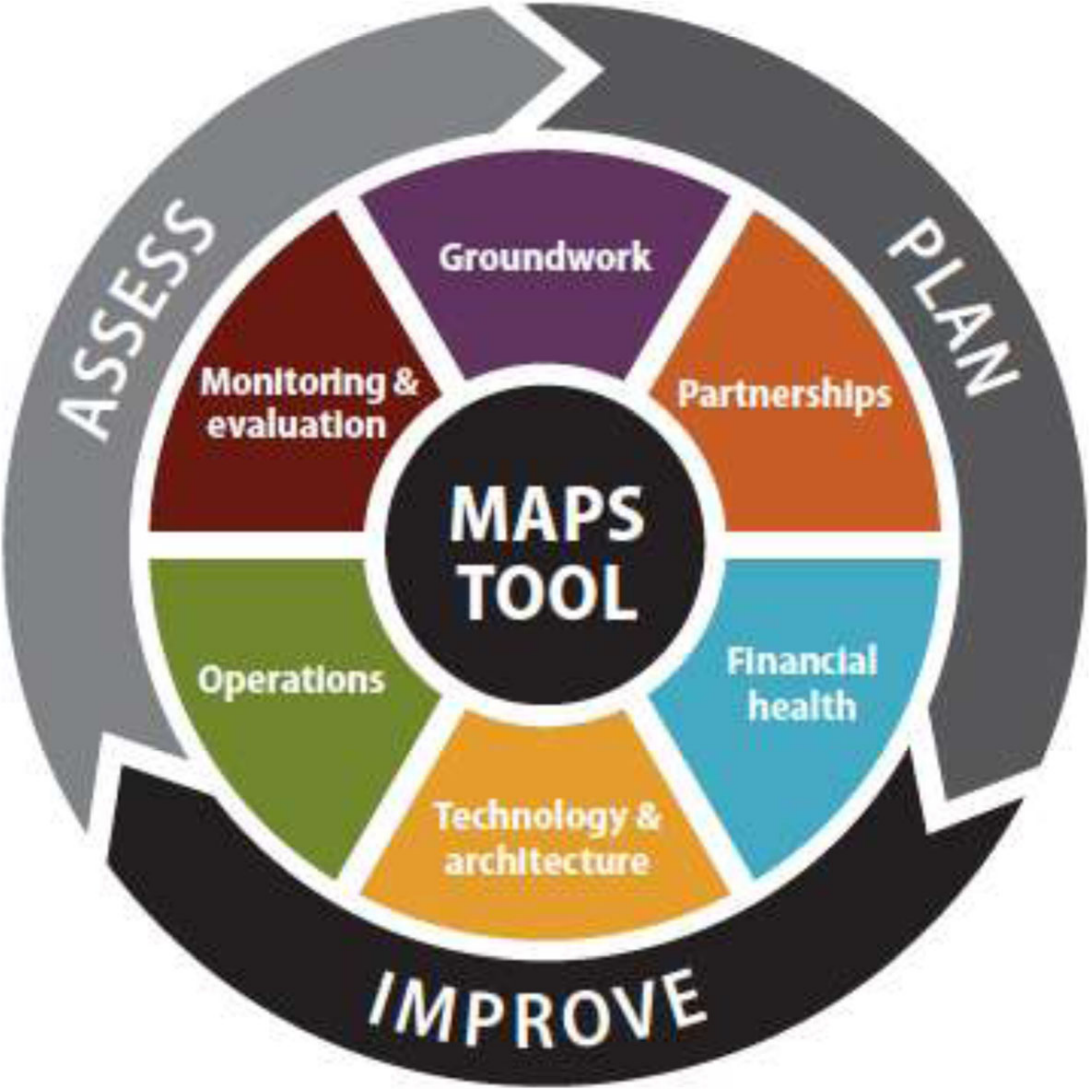
Conceptual model of the MAPS Toolkit to measure digital health project maturity across six axes [2]

In 2015, the World Health Organization (WHO), the United Nations (UN) Foundation and Johns Hopkins University Global mHealth Initiative jointly developed the mHealth Assessment and Planning for Scale Toolkit (MAPS), which includes a self-scoring rubric to examine digital health project maturity across six axes (Fig. 1) [2]. Scoring the technology, architecture and the scientific underpinnings of the digital health solution allows the identification of strengths and weaknesses, while structural integrity at scale can be assessed in terms of operations and financial health. And finally, external partnerships and linkages are examined, exploring the degree to which the digital solution benefits other stakeholders of the health ecosystem [2].

### Practical experience of scaling up digital health innovations

**Fig. 2:**
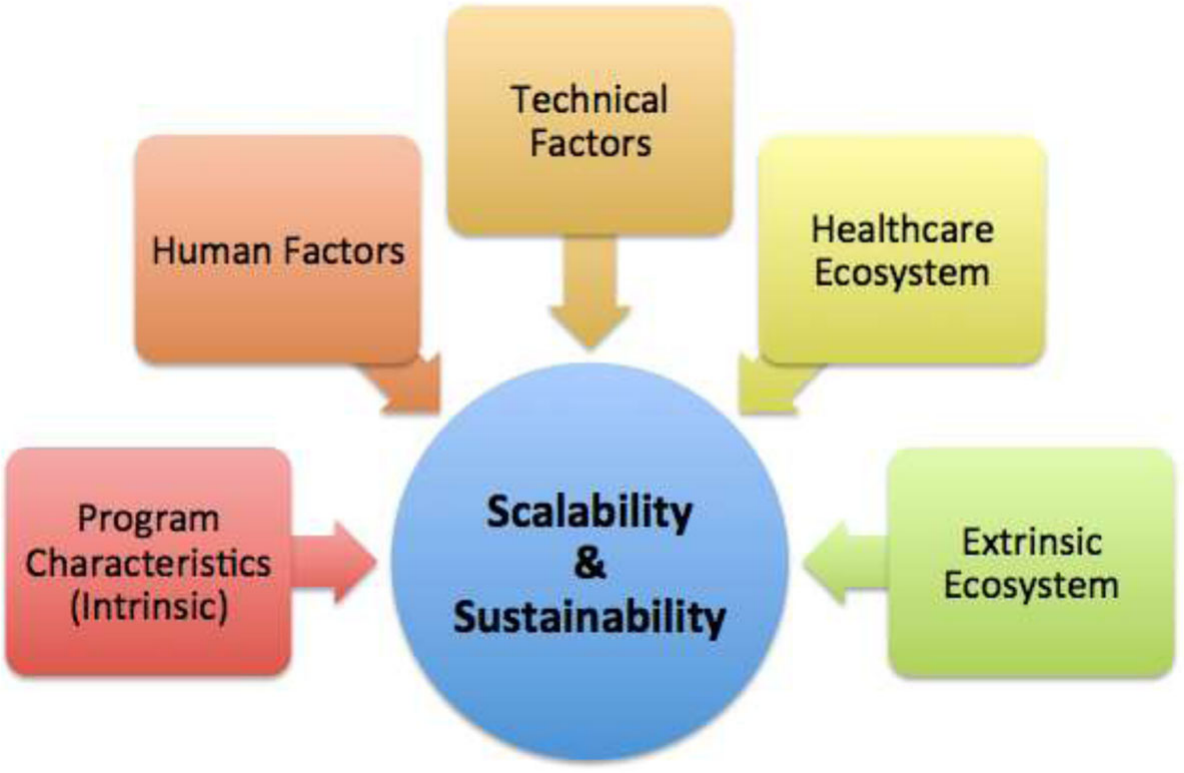
Practical considerations for scaling digital health initiatives in LMICs

Practical experience from real-life case studies of scaling and sustaining digital health initiatives in LMICs has highlighted five critical focus areas: programme characteristics, human factors and technical factors, as well as the features of the healthcare ecosystem and broader extrinsic ecosystem within which they operate (Fig. 2). Each of these elements should be considered individually when planning for scale, but must also form an interlinked system.

#### Programme characteristics (intrinsic)

User-centred programme design is an essential principle for successful digital health initiatives, particularly when planning for scale and integration. The Principles for Digital Development, describing best practices for scale, specifically outline the importance of ensuring that innovations are contextually appropriate and valuable for all stakeholders: end-users and policymakers as well as investors (Table 2) [3]. Given that the end-users in most government-led programmes (frontline health workers, community-based clinicians, etc) are seldom involved in the financial decision-making around which information system tools will be purchased or adopted, it is not uncommon for them to be excluded from the design and development process [3]. A Ghana-based telemedicine initiative was showcased as a successful example of scaling digital health based on end-user insights [4]. The initiative relied on the utilisation of mobile phones by community health workers (end-users) in remote communities and districts. User-based design was a critical success factor resulting in sustained levels of uptake and use. This enabled the telemedicine services to be expanded and rolled-out nationwide in Ghana, providing measurable benefits to the health system, including reductions in unnecessary hospital referrals.

**Table 2:** Principles for Digital Development, a consensus statement of best practices (2014) [3]

• Design with the user	
• Understand the existing ecosystem	
• Design for scale	
• Build for sustainability	
• Be data-driven	
• User open standards, open data, open-source and open innovation	
• Reuse and improve	
• Address privacy and security	
• Be collaborative	

A frequently neglected opportunity within digital programme design for scale is the capture and utilisation of real-time data; ensuring that end-users, supervisors, health system managers and policy makers have access to and are trained to use real-time information. This can improve outcomes by empowering users to track their own performance, motivating health workers by peer comparisons or distance-from-targets, or by making the incentivisation process transparent. In most cases, this is a major shift from current monthly or annual reporting, and may require changes to policy, training and accountability. Without adequate decentralisation that allows for local decision-making based on real-time data, this opportunity may be missed. The CommCare platform [5] enables immediate data entry and real-time monitoring, which significantly benefits frontline healthcare workers: it reduces average data entry timelines and allows healthcare workers to immediately monitor their adherence to protocols, or identify where improvements were needed [6, 7]. As well as improving performance, analysis of real-time data can be used to justify scaling and additional investment. In Bangladesh, the Open Smart Register Platform is used by the Ministry of Health to digitise paper registries in maternal and child health [8, 9]. This system allows synchronisation of data on individual patients, reduces duplication, and can send key data directly to the national health information system. Local and national level supervisors use real-time dashboards for supportive supervision and to examine and compare health centre performance, which can contribute to refining performance while taking programmes to scale.

#### Human factors

To achieve scale, it is critical that end-users are also motivated, trained and prepared to fully embrace and utilise any digital health solution. Effective, sustainable support systems and training, as well as clarity on roles and accountability for decision-making and supervisory structures are essential components. Often, improvements in health systems can fail to provide adequate training for the supervisors of frontline workers equipped with new technologies, potentially excluding them from an information flow that was built around multiple layers of quality control and oversight. Experience has shown that any training should be easy to replicate and to roll-out at large for end-users across locations.

A highlighted example of effective end-user training was the community-based hypertension improvement project (ComHIP) in the Eastern Region of Ghana, which offered training to licensed chemical sellers in the community to screen for hypertension and provide adequate health education and information on hypertension to their clients [10, 11]. This model, underpinned by a comprehensive digital health platform, maximises opportunities for screening and diagnosis of hypertension in a peri-urban area of sub-Saharan Africa and brings chronic disease care closer to the community [11]. The digital health platform connects the licensed chemical sellers with community health workers and health service providers at referral sites. It also empowers patients to take more responsibility in the management of their own health. This innovative programme is an example of how digital health can help to alleviate the burden on sometimes overloaded primary healthcare systems [10, 11]. Another approach to address and overcome the need for training and support for new digital health initiatives is the repurposing of existing, familiar technologies. The M-TIBA mobile health wallet in Kenya was successfully built upon the existing M-PESA system for transferring money across digital networks [12]. The simple mobile payment system was leveraged to enable users to save and pay for healthcare services. As M-PESA was already accepted and familiar throughout Kenya, it facilitated rapid uptake and scaling of the M-TIBA mobile health wallet. This initiative also provides an example of the benefits of private sector involvement with respect to factors such as sustainable funding, technical expertise and operational efficiency.

A persistent challenge in resource-limited contexts is that the introduction of digital innovations may add increased scrutiny of performance and efficiency, which may also uncover institutional dysfunctions. Whether fuelled by inertia, resistance to change, conflicting priorities, lack of training, absence of engagement or lack of clarity in roles and responsibilities, it is important to consider such factors and understand them, to adequately plan for scale. Finally, the importance of human resource capacity cannot be ignored as programmes plan to scale-up. Qualified technical staff in health informatics, information sciences, statistical analysis and technology management are essential, but often in short supply. Unless educational institutions support the development and training of more skilled personnel, and pre- as well as in-service training is introduced, this may remain an obstacle to scale.

#### Technical factors

Technical factors intrinsic or external to the digital health initiative are critical components of scalability and sustainability. One key ‘technical’ feature to consider may be simplicity in function. As opposed to complex technologies with multiple functions, simple systems may be easier to scale, as documented in a recent global survey of frontline health worker technologies [13]. Whether this is due to technical challenges or difficulty to obtain buy-in for a more complicated solution is unclear. Gaining stakeholder acceptance around complex technologies also seems to be a challenge in policy and budgetary environments that tend to be risk-averse. Simple solutions tend to be less dependent on the extrinsic environment, often relying on more robust cellular channels such as short message service (SMS), interactive voice response (IVR), unstructured supplementary service data (USSD) or voice, rather than internet or data. The *mDiabetes Project* in Senegal, providing a simple daily SMS with health advice to patients with diabetes [14], and *BBC Mobile Kunji* [15], an audio visual job aid to help community health workers in Bihar to counsel families, were highlighted as case studies of digital health innovations enjoying rapid scale-up thanks to their inherent simplicity.

Interoperability is among the key focal areas of a new global partnership known as the Health Data Collaborative (HDC) and the Principles for Digital Development [3]. Interoperable systems can ‘speak’ to one another, and more importantly, share information to avoid duplication, reduce burden on healthcare workers and clients, and magnify impact through collaboration. Frustrated by the proliferation of separate projects that were not sharing data, nor integrated within the health system, the Ministry of Health of Uganda issued a moratorium on mHealth projects in the country in 2012 [16]. This decision was a turning point for the information and communication technology (ICT) for development agenda, realising that the fragmented landscape was not unique to Uganda, but reflected the reality in many countries. Lightly-funded, often well-intended innovation projects had been operating in siloes, addressing single health problems in a vertical way, without making lasting contributions to the overall health system. As a result, investments in ‘global goods’ that help facilitate interoperability have increased, from the development of terminology services like the Columbia International eHealth Laboratory (CIEL) Concept Dictionary [17], to open-source health information exchange (HIE) architecture^1^, that helps create shared services (e.g. facility registries, worker registries, patient identifiers) [16, 18].

Additional challenges to scale identified by the cases studies presented were lack of software interoperability and adaptability to changing requirements, as well as considerations of data storage, access and confidentiality. Smaller-scale systems tend to be hard-coded for a specific project or demonstration pilot, unable to significantly increase the number of users without frequent dysfunction. It was widely agreed that extendibility and adaptability of digital health solutions also contributes to further roll-out and scale. The use of commonly available platforms such as CommCare, OpenSRP, OpenMRS, and OpenDataKit [5, 19–21] is both encouraging interoperability and providing a framework for easy adaptation. The CommCare example has proven to be both beneficial and efficient, demonstrated by the multiple health programmes that have now used and adapted the platform for roll-out and scale [22]. The platform has now released HIE functionality which will further enable different CommCare deployments to be connected and share data [5].

#### Healthcare ecosystem

As important as programme, human and technical factors is the environment in which the digital health initiative must function at scale. This includes financial support that enables long-term sustainable operations, and regulatory standards and frameworks that ensure compliance with national health guidelines and strategies. In many settings the absence of strong digital health policies and standards make it difficult to design solutions for scale. Close collaboration with government stakeholders and other policy makers can be useful to help inform emerging digital health policies, and to provide information to technical teams about regulatory changes on the horizon. In the example of the Uganda mHealth moratorium, the government chose to align the goals of digital health with a broader national health strategy [16]. The ComHIP programme in Ghana on the other hand, provides an example of a programme that addresses unmet needs in managing chronic diseases in LMICs by leveraging existing technologies that do not require policy engagement [11]. For example, ComHIP utilises mobile phones to empower patients by sending daily reminders for follow-up appointments, medication adherence, and nutritional health information.

Another extrinsic consideration identified as important among the case studies was the need for sustainable financing. The funding plan for a digital health programme must be clear from the outset, with a path towards sustainability that may require public-private partnerships, or that may include contingencies so that it can evolve over time. It is estimated that around 60% of healthcare financing in Africa is derived from private sources, with specific contributions and funding models varying according to the healthcare contexts in different countries [23]. The Ghana telemedicine services benefited from a public-private sector partnership that provided both funding and expertise during the set-up and pilot phase, after which local ownership was fully established [4]. The initiative has now reached a stage where the operational and maintenance costs are covered by the Ghana Health Service and the roll-out of the national roadmap for telemedicine is fully in the hands of the local health authorities [4]. Similarly, in South Africa, the MomConnect programme began as a public-private initiative, largely subsidised by donors and private sector partners [24]. In 2015, the programme was integrated into the National Department of Health, and it is now offered to all South African pregnant women as a free government service.

Contingency planning is another important factor to consider for scaling digital health programmes. Healthcare priorities in LMICs may change rather rapidly, for example when new pathogens emerge, or old diseases spiral out of control. The recent example of Ebola highlights how a diversion of healthcare resources (funding and manpower) is needed to enable a rapid response. This learning, however, did provide an important opportunity for the global digital health community to examine its readiness and capacity to respond to emergencies. Poor communications and local electricity infrastructure made it quite challenging to maximise the real potential for digital tools in managing a widespread response [25]. Very complex communication and information chains made the task of unifying information streams nearly impossible. An outstanding example of an open-source, built-for-scale platform emerged during and after the Ebola crisis as mHERO [26], which combines IntraHealth’s Integrated Human Resource Information System (iHRIS) system for workforce management, the UN Children’s Fund (UNICEF)‘s RapidPro text messaging platform, the District Health Information System (DHIS2), and the Open health information exchange (OpenHIE) backbone. mHERO specifically strives to use robust, existing solutions to support information and communication needs across West Africa and beyond [26]. Similarly, the roll-out of the electronic Tool to Improve Quality of Health (eTIQH) in Tanzania and Mali benefited from a combined top-down and bottom-up approach for reaching scale [27]. The results from one country convinced the health authorities in the other country to take on eTIQH, and scale it within their country.

#### Extrinsic ecosystem

Practical experience has identified the reliability and bandwidth of networks, as well as availability of electricity to charge devices as key extrinsic limiting factors affecting scaling of digital health projects in LMICs. Availability of high-quality hardware was also identified as a local ecosystem constraint limiting scale-up, which may benefit from funding from the private sector. Technical dimensions must be considered at the start of a programme, since retrofitting a technology for scale is extremely difficult. Building for scale is neither inexpensive nor easy, but a robust architecture can allow a system to perform as smoothly with 50, 50,000 or 500,000 users. Data management and storage, assurance of patient data security, and reliable offline and back-up systems suited to LMICs must all be considered during the design phase of a digital solution. The CommCare mobile health platform for frontline health workers is an example of such architecture, which has scaled to > 50,000 mobile users [28]. The system has been specifically designed for LMIC use at scale and includes a robust infrastructure, patient security options, and offline usage to support frontline health workers in remote locations. Offline capacity with the ability to store and delay the upload of data is an important feature of digital solutions in LMICs, as it allows users to enter data and make use of the system even when network connection is unreliable or not available.

## Conclusions

The recommendations in this manuscript have been collated based on case studies from programmes on the ground and from experts, who were willing to discuss the successes and failures of their work in digital health within LMICs. As an emergent space in global development, specific guidelines and best practices for digital health will likely change rapidly – but the core principles identified across these five domains are likely to hold true. From the outset, close engagement of all stakeholders and a commitment to resolving pain points in existing workflows must be of paramount importance. Programme readiness for scale can be honed, with consideration of the technical capacity for increasing the number of users, adaptability, and connectivity to national and parallel systems. Digital health innovators must actively advocate for investments in the external elements required for scale-up. Gaps in policies and standards or an unstable infrastructure will certainly hinder scalability of digital health innovations.

Close partnership among stakeholders, from inception through to scale-up is important. By leveraging insights and expertise from different end-users, technology providers, health policy makers and healthcare programme managers, we can come closer to successful scaling of digital health solutions in LMICs to address unmet needs. The scaling process needs to be dynamic and flexible, and allow adaptations for changes in human or technology needs of the system in which the programme is operating. The private sector has an important role to play in initiating and scaling digital health initiatives, dependent on the healthcare policy environment [23]. Where resources and expertise are overstretched in the public sector, the private sector may be able to offer funding, expertise and support with infrastructure. In each case, clarity on nature and duration of the partnership is critical, to ensure digital health initiatives can be scaled and sustained over the long-term [23].

**Fig. 3:**
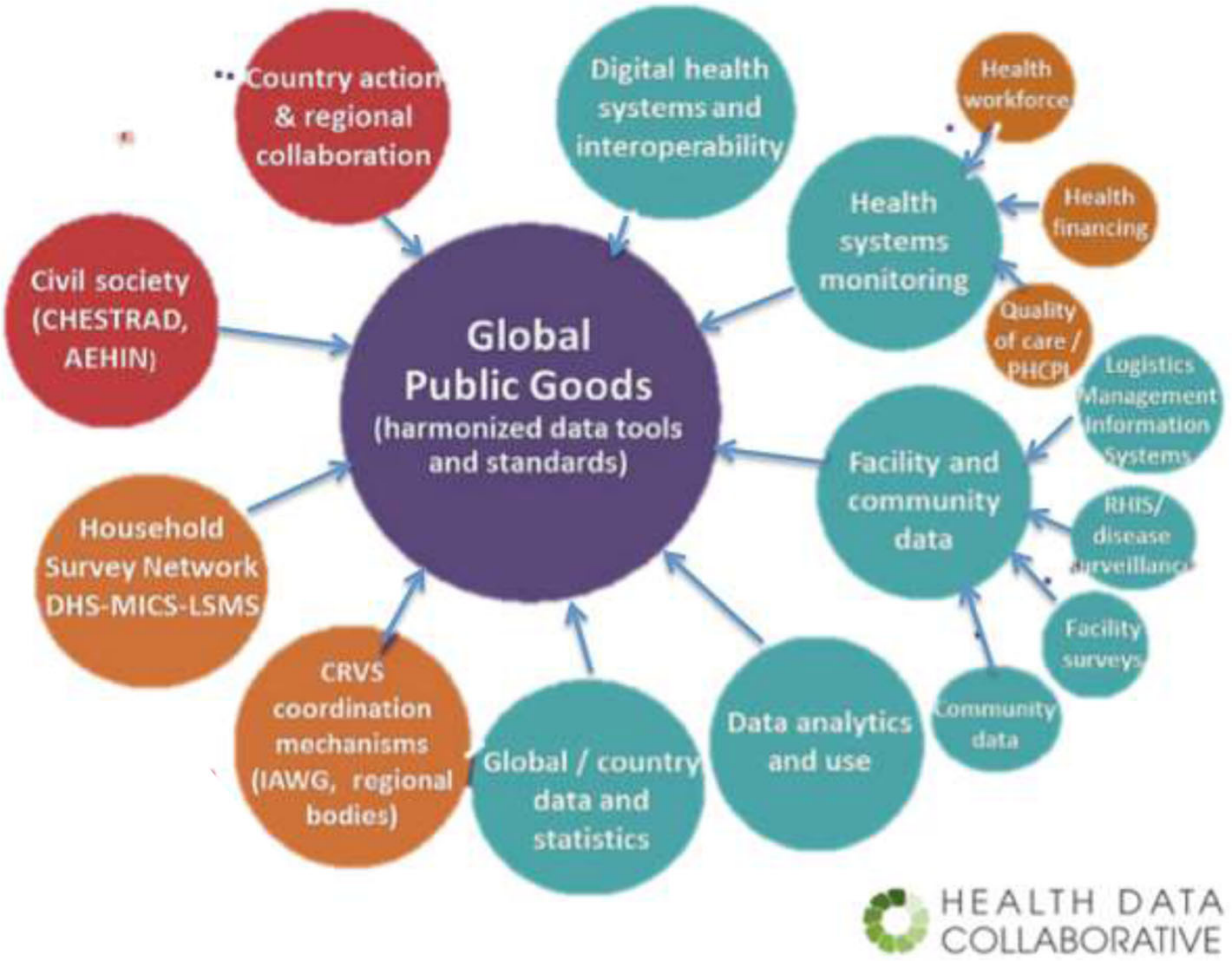
Working groups of the Health Data Collaborative (2016) [34]

Much like a seed planted to grow, the best digital health innovations may remain stifled unless conditions of fertility are optimal. On a global scale, several initiatives are ongoing to support scaling of successful digital health initiatives. In 2012, the WHO-International Telecommunications Union (ITU) toolkit for national eHealth strategies described optimal conditions for scaling, including the enabling policy environment, as well as the needs in human resources capabilities and technology infrastructure [29]. Many of these factors fall outside the influence of individual projects, such as the stability of the national electrical grid, the availability of cellular phone coverage in remote parts of the country or the need for strong government leadership and commitment to digital health. However, project implementers are important stakeholders to influence and drive this agenda forward at the national level. For example, in South Africa, India, Rwanda and Uganda, the Ministry of Health or the Central Government have convened Advisory Committees, including members of the digital health ecosystem, to advocate for improvements in the enabling environment for digital health. The HDC, mentioned earlier, and recent multilateral initiatives like the Digital Impact Alliance (DIAL) and Digital Square, which include international agencies, governments, philanthropies, donors and academics aim to improve health data through shared investments in ‘global goods’, to accelerate progress and scale-up of successful digital health solutions [30–33]. These global goods range from policy guidelines to backbone technologies, and are meant to assist Advisory Committees and Government Agencies in launching, scaling, and sustaining digital health innovations. The HDC hopes to address the existing lack of donor coordination by allowing shared investments in specific countries and global goods. By strengthening country information systems, the HDC aims to contribute to better decision-making and, ultimately better health [31]. The different working groups of the HDC will be developing standards, indicators and other tools recommended for countries to collect, analyse and use health data (Fig. 3) [34]. This may be an important step forward for facilitating the scalability of digital health solutions. Finally, in the beginning of 2017, the Broadband Commission for Sustainable Development launched the report of its digital health working group, chaired by the Novartis Foundation and Nokia [35]. The report explores in depth one of the essential steps to realise the full potential of digital health, namely sustained and committed leadership.

Over the next decade, the trajectory of digital health towards scale will certainly continue to evolve and accelerate. In the past 10 years, we have witnessed a mobile phone revolution that has connected the most distant communities. If Moore’s 1965 law, which predicts the doubling of computer processing capacity every 18–24 months [36], holds true for digital health, we will continue to observe rapid increases in internet access and potential digital solutions to health problems. Digital innovations will continue to extend connectivity to the last mile of populations and it is difficult to anticipate how health systems will absorb and mainstream these technologies. In addition to the further democratisation of health information, the ubiquity and lower cost of technology may make the adoption and scale-up of innovation easier. Although not discussed enough, the possible risks associated with digital health innovations need to be considered. For example, the rapid spread of misinformation through social media or unscrupulous use of health information to promote self-medication make it essential for implementers to take responsibility to ‘first do no harm’. Finally, it remains imperative that the global health community doesn’t grow enamoured with technology as an end in itself, but insists that digital innovation is a means to an end focused on solving problems, improving health, and saving lives. Only by realising the full potential of digital health, and providing appropriate leadership, can we accelerate the achievement of sustainable development goal 3, Health for All.

## Abbreviations

CIEL: Columbia International eHealth Laboratory
DHIS2: District Health Information System
DIAL: Digital Impact Alliance
eTIQH: electronic Tool to Improve Quality of Health
HDC: Health Data Collaborative
HIE: Health information exchange
ICT: Information and communication technology
IHRIS: Integrated Human Resource Information System
ITU: International Telecommunications Union
IVR: Interactive voice response
LMIC: low and middle income countries
MAPS: mHealth Assessment and Planning for Scale Toolkit
OpenHIE: Open health information exchange
PATH: Program for Appropriate Technology in Health
SMS: Short message service
UN: United Nations
UNICEF: United Nations Children’s Fund
USSD: Unstructured supplementary service data
WHO: World Health Organization

## Footnotes

1 For more details on HIE, the authors recommend the Open Health Information Exchange community (OHIE.ORG)
